# Association between chronic hepatitis B virus infection and stroke risk: a propensity score-matched analysis

**DOI:** 10.1017/S095026882610154X

**Published:** 2026-05-05

**Authors:** Amir Yahav, Doaa Ryan, Nael Tuma, Anat Arbel, Nili Stein, Eitan Auriel, Walid Saliba

**Affiliations:** 1 Ruth and Bruce Rappaport Faculty of Medicine, Technion Israel Institute of Technology, Haifa, Israel; 2 Department of Community Medicine and Epidemiology, Lady Davis Carmel Medical Center, Haifa, Israel; 3 Northern District Health Bureau, Israel Ministry of Health, Nof Hagalil, Israel; 4 Infectious Diseases Unit, Lady Davis Carmel Medical Center, Haifa, Israel; 5 Statistical Unit, Lady Davis Carmel Medical Center, Haifa, Israel; 6 Department of Neurology, Rabin Medical Center Beilinson Hospital, Petach Tikva, Israel; 7 Gray Faculty of Medical & Health Sciences, Tel Aviv University, Tel Aviv, Israel

**Keywords:** HBsAg, HBV, hepatitis B virus, hepatitis D virus, intracerebral haemorrhage, ischaemic stroke, stroke

## Abstract

Evidence on the association between chronic hepatitis B virus (HBV) infection and stroke is limited, inconsistent, and confined predominantly to endemic regions in Asia. This study investigated the association between chronic HBV infection and stroke using data from the largest healthcare provider in Israel. All individuals aged 20 and older who were tested for hepatitis B surface antigen (HBsAg) between 2005 and 2023 were identified. Newly diagnosed HBV patients (HBsAg-positive) were propensity scorematched to non-HBV subjects (HBsAg-negative) in a 1:4 ratio and followed for stroke occurrence through 2024. The study included 20 544 HBV patients and 82 176 matched controls. Overall stroke was diagnosed in 472 HBV patients and 1 717 controls (incidence rates: 2.13 vs. 1.94 per 1 000 person-years). Hazard ratios were 1.09 (95% CI, 0.98–1.22) for overall stroke, 1.01 (0.89–1.14) for ischemic stroke, and 1.82 (1.35–2.45) for intracerebral hemorrhage (ICH). Ischemic stroke risk was specifically increased in younger individuals and females (p-for-interaction = 0.006 and 0.079, respectively). Results remained consistent when excluding patients with prior stroke. Exploratory analysis suggested hepatitis D coinfection is associated with increased ICH risk. In conclusion, chronic HBV infection was associated with significantly increased ICH risk, with subgroup-specific increases in ischemic stroke risk.

## Introduction

Stroke is a leading cause of death and disability worldwide, with its burden rising over the past three decades [1, 2]. Globally, ischaemic stroke accounts for approximately 80–85% of all strokes, while intracerebral haemorrhage (ICH) comprises most of the remainder [1, 2]. The burden of stroke varies geographically, being highest in middle- and low-income countries [1, 2]. While traditional risk factors such as hypertension, diabetes, and dyslipidaemia account for a significant proportion of stroke cases, they do not fully explain the observed variation in stroke incidence [1, 2]. Emerging evidence suggests that various infections, including both acute and chronic conditions like herpes zoster and hepatitis C virus (HCV), may also play a role in stroke pathogenesis [3, 4]. However, the impact of hepatitis B virus (HBV) on stroke risk is yet to be determined [5].

HBV is a hepatotropic virus transmitted via blood and bodily fluids, affecting an estimated 3.3% of the global population [6]. Chronic infection develops in about 90% of infants infected perinatally but only 5%–10% of adults [6]. Like stroke, HBV is more prevalent in low-income countries, mainly due to low vaccination coverage and perinatal transmission [6]. Chronic HBV is a leading cause of liver cirrhosis and hepatocellular carcinoma, the most prevalent liver cancer [6].

A biological link between chronic HBV and stroke may involve an interplay between chronic systemic inflammation and liver damage. Persistent inflammation may promote atherosclerosis and endothelial dysfunction, increasing the risk of ischaemic events [7], while also weakening vessel walls and predisposing to haemorrhage [8]. Liver dysfunction can further contribute to coagulopathy and thrombocytopenia, increasing haemorrhagic risk [9], but may also lower lipid levels, potentially reducing ischaemic risk [1].

Evidence regarding the association between HBV and stroke is limited, inconsistent, and confined to endemic regions in Asia. A large cohort study from China reported no association between HBV and overall stroke or ischaemic stroke, but noted an increased risk for ICH [5]. Conversely, two other studies from Korea [10] and Taiwan [11] found a decreased risk for ischaemic stroke among HBV-infected patients. Variations in the adjustment for potential confounders, methods of HBV exposure ascertainment, population characteristics, and the presence of liver dysfunction may explain these heterogeneous results. To our knowledge, no study has investigated the association between the hepatitis D virus (HDV), which co-infects individuals with HBV and is considered the most severe form of viral hepatitis [12].

Given these uncertainties, we aimed to assess the relationship between chronic HBV infection and the risk of stroke, as well as its main subtypes, leveraging the comprehensive and long-standing database of Clalit Health Services (CHS).

## Materials and methods

### Source of data

This retrospective cohort study is based on data from the CHS database, the largest health maintenance organization (HMO) in Israel. CHS provides inclusive health care for more than half of the Israeli population (~4.9 million). Health care coverage in Israel is mandatory according to the National Health Insurance Law (1995) and is provided by four groups akin to not-for-profit HMOs, which are charged with providing a broad package of benefits stipulated by the government. The four HMOs are both health-care insurers and providers, thus financing and supplying health services. Membership in a specific HMO is voluntary, and members can freely switch to another HMO. All members of the different HMOs have a similar health insurance plan and similar access to health services, including low medication co-payments. CHS maintains a database that receives data from multiple sources, including records of primary care physicians, community specialty clinics, hospitalizations, laboratories, and pharmacies. A registry of chronic disease diagnoses is compiled from these data sources. Diagnoses are captured in the registry by diagnosis-specific algorithms, employing International Classification of Diseases, Ninth Revision (ICD-9) code reading, laboratory test results, and disease-specific drug usage. A record is kept of the data sources and dates used to establish the diagnosis, with the earliest recorded date, from any source, considered to be the defining date of diagnosis. Designed for the purposes of administrative and clinical management, the database is available for clinical studies. Relevant high-quality studies have been conducted based on data from the CHS database [13, 14].

### Study population and design

The population of this retrospective cohort study included patients who tested positive for HBsAg (hepatitis B surface antigen), which constitutes the exposed group, and a propensity score-matched comparative group who tested negative for HBsAg, which constitutes the unexposed group. To select the study population, we first identified all adults 20 years or older from the CHS database who had undergone an HBsAg test with valid results between 1 January 2005 and 31 December 2023. Individuals with a prior positive HBsAg test were excluded. For the exposed group, the index date was defined as the date of the first documented HBsAg-positive test. For the unexposed group, the index date was defined as the date of the first documented HBsAg-negative test among individuals with only negative test results.

From the eligible HBsAg-negative subjects, we randomly selected a subset of 500 000 subjects for computational efficiency. We used logistic regression to calculate the predicted probability (propensity score) of being positive for HBsAg for both this subset and the exposed subjects using various factors, including demographic variables, baseline comorbidities, medication use, and medical services use. Patients in the positive HBsAg group were propensity score-matched to patients in the negative HBsAg group in a 1:4 ratio using a greedy matching algorithm with a maximum acceptable difference of 0.001 in the propensity score between matched groups (calliper width of 0.001).

The identified HBV patients (exposed group) and their matched comparative non-HBV controls (unexposed group) were followed from the index date until the first occurrence of study outcome (ICH or ischaemic stroke), death, or end of follow-up (31 December 2024), whichever came first.

### Definition of outcome and exposure

The study outcomes included overall stroke, defined as a composite of ischaemic stroke and ICH, as well as each of these components examined separately. Ischaemic stroke was defined as a primary discharge diagnosis with ICD-9 codes 433.x1, 434.x1, and 436. While these codes reliably ascertain acute ischaemic stroke events as a whole [15], they do not permit specific subtyping (e.g., lacunar, large-artery atherosclerosis, or cardioembolic), which requires additional neuroimaging and clinical information [16].

ICH was defined as a primary discharge diagnosis with ICD-9 code 431. Previous research by our group on the CHS database revealed a positive predictive value of 86.4% for the diagnosis of ICH using ICD-9 code 431 [13].

Chronic HBV infection was defined as having at least one positive serological HBsAg test result, obtained from either hospital or community settings. Clinical diagnosis of chronicity requires HBsAg positivity for at least 6 months. However, HBsAg positivity alone is a recognized pragmatic approach to define chronic HBV infection in population-based research [5, 10]. This approach reflects the low prevalence of acute HBV infection among adults in the general population, particularly in regions with low HBV incidence, such as Israel, where most HBsAg-positive adults are chronic carriers rather than acutely infected [6, 17].

### Study variables

For each patient, we extracted baseline variables from the CHS computerized database. These included sociodemographic information, such as age, sex, population sector (Jewish or Arab), socioeconomic status (SES) based on the SES scores of the clinic neighbourhood as defined by the Israeli Central Bureau of Statistics, and district in the CHS. SES and district had missing values (7.9% and 0.3% respectively); hence, these variables were treated as categorical variables that include a category of missing values. In addition, we gathered data on cardiovascular risk factors and comorbidities, including: smoking status, hypertension, diabetes mellitus, obesity, hyperlipidaemia, family history of cardiovascular disease, alcohol abuse, drug abuse, cardiac valvular disease, intermittent peripheral vascular disease (PVD), carotid stenosis, aortic aneurysm, atrial fibrillation, ischaemic heart disease, congestive heart failure, prior stroke, chronic renal disease, chronic obstructive lung disease (COPD), and malignancy. We also included the following medications: antiplatelets, anticoagulants, and statins. Furthermore, we collected data on health behaviour markers and medical service utilization, including performance of faecal occult blood test and influenza vaccinations within 2 years before cohort entry, pneumococcal vaccinations within 5 years before cohort entry, hospital admission within the year before cohort entry, and the number of visits to a primary care physician within the year before cohort entry. We also retrieved baseline alanine aminotransferase (ALT) levels and the status of hepatitis D (HDV) virus coinfection, which was defined by the documentation of hepatitis D antibodies or RNA PCR serological tests.

### Statistical methods

All statistical analyses were performed using IBM SPSS Statistics, version 28.0 (IBM Corp., Armonk, NY, USA) and SAS software, version 9.3 (SAS Institute Inc., Cary, NC, USA). Descriptive statistics were used to summarize study variables according to HBV exposure status. Continuous variables with normal distributions are presented as means ± standard deviation (SD), and categorical variables are presented as frequencies and percentages. To evaluate the balance of baseline characteristics between the HBV and non-HBV groups in both the full cohort and the propensity score-matched sample, we used the standardized mean difference (SMD). An SMD of 0.1 or less indicates a negligible difference in the measured variables between groups [18]. To assess the association between HBV and the study outcomes, we used a Cox proportional hazards regression model stratified by the matching indicator. Results are reported as hazard ratios (HRs) with 95% confidence intervals (CIs). Potential effect modification by sex and dichotomized age (at the median) was evaluated by including interaction terms in the Cox regression models.

We performed three sensitivity analyses; First, to estimate the association with the first event of stroke, we restricted the analysis to subjects without a prior stroke diagnosis. Second, we repeated the analysis after excluding participants who had ever been diagnosed with HCV. Additionally, we limited the analysis to confirmed chronic HBV cases, defined as at least two positive HBsAg tests obtained ≥6 months apart.

We also evaluated the association between HBV infection and study outcomes in two specific contexts: in individuals coinfected with HDV, and in those with HBV infection accompanied by elevated ALT levels (> 40 U/L) as a marker of disease activity.

## Results

**Figure 1.**
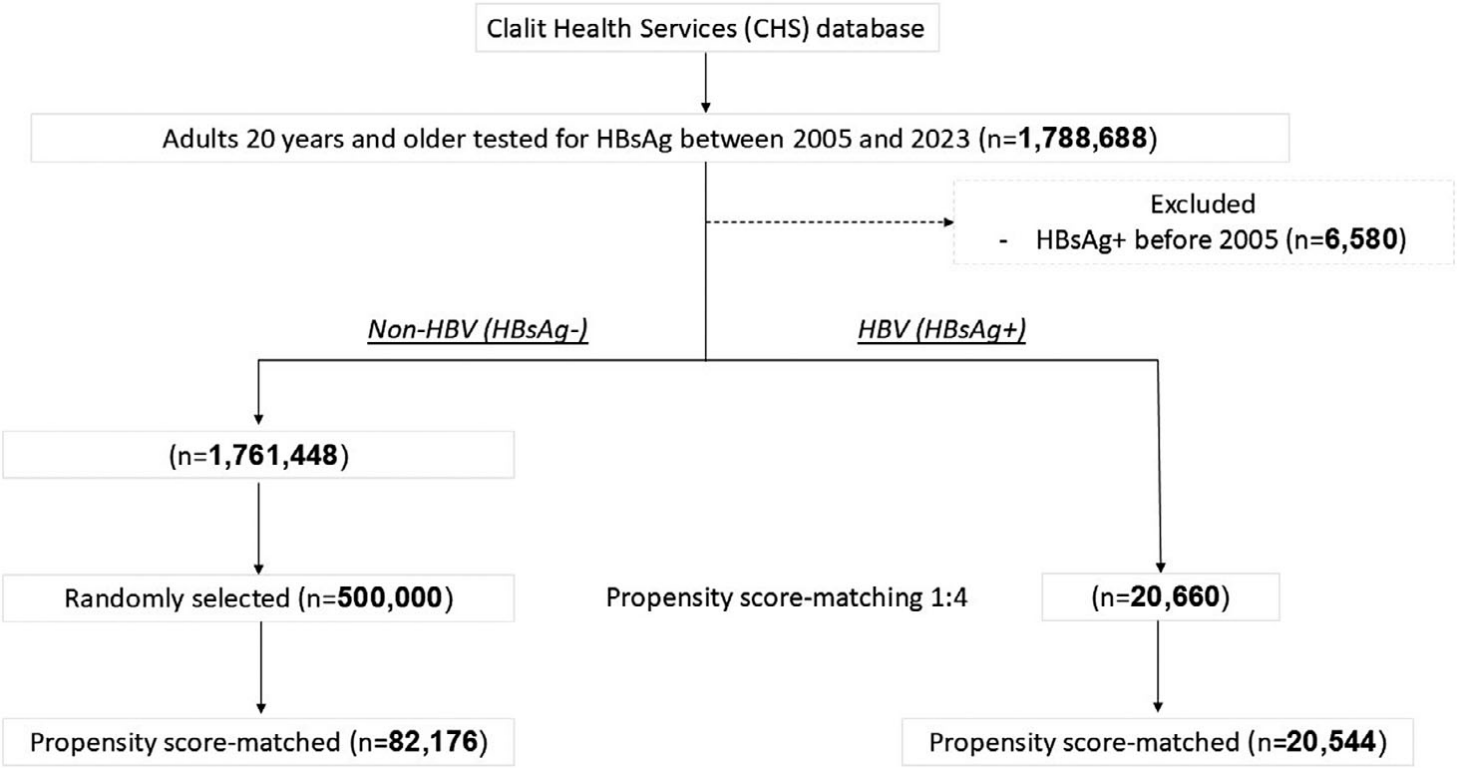
Study flow diagram depicting the selection of the study population. Abbreviations: HBV, hepatitis B virus; HBsAg, hepatitis B surface antigen; HCV. Figure 1. long description.

Overall, 1 782 110 subjects were eligible for inclusion in the study, comprising 20 660 HBV and 1 761 448 non-HBV subjects. From the non-HBV group, 500 000 subjects were randomly selected and used for matching. Using this subset, a propensity score-matched sample of 20 544 HBV and 82 176 non-HBV subjects was selected and included in the analyses. A flow chart for the selection of the study population appears in Figure 1.

Table 1 shows a comparison of baseline characteristics between the HBV and the non-HBV groups before and after matching. Before matching, subjects in the HBV group were older, more likely to be men, and more likely to be from a low SES background. They were also more likely to have diabetes, to take antiplatelets, and to perform faecal occult blood tests. After matching, all differences between the HBV and non-HBV groups were balanced with SMDs less than 0.1.

**Table 1.** Baseline characteristics of the study population before and after propensity score-matching Table 1. long description.

Variable	Before matching			After matching		
	HBV(*n* = 20 660)	Non-HBV(*n* = 500 000)	SMD	HBV(*n* = 20 544)	Non-HBV(*n* = 82 176)	SMD
Age at index date (y)	44.6 ± 15.8	39.4 ± 17.5	0.31	44.6 ± 15.8	44.2 ± 17.9	0.02
Sex (male)	11 620 (56.2)	185 566 (37.1)	0.39	11 539 (56.2)	46 625 (56.7)	0.01
**Population sector**						
Jewish	14 775 (71.5)	396 633 (79.3)	0.18	14 688 (71.5)	61 149 (74.4)	0.07
Arab	5 887 (28.5)	103 367 (20.7)	0.18	5 856 (28.5)	21 027 (25.6)	0.07
**Socioeconomic status** (SES)^a^						
Low	9 668 (46.8)	181 969 (36.4)	0.21	9 609 (46.8)	37 712 (45.9)	0.02
Middle	7 635 (37.0)	226 802 (45.4)	0.17	7 598 (37.0)	30 046 (36.6)	0.01
High	887 (4.3)	52 690 (10.5)	0.24	886 (4.3)	3 635 (4.4)	0.00
**District^b^**						
A	1 983 (9.6)	54 215 (10.8)	0.04	1 975 (9.6)	7 573 (9.2)	0.01
B	1 920 (9.3)	46 705 (9.3)	0.00	1 912 (9.3)	8 072 (9.8)	0.02
C	1 923 (9.3)	56 718 (11.3)	0.07	1 905 (9.3)	7 564 (9.2)	0.00
D	3 908 (18.9)	85 297 (17.1)	0.05	3 889 (18.9)	15 903 (19.4)	0.01
E	2 771 (13.4)	67 422 (13.5)	0.00	2 758 (13.4)	11 000 (13.4)	0.00
F	3 165 (15.3)	59 780 (12.0	0.10	3 138 (15.3)	12 442 (15.1)	0.01
G	2 655 (12.8)	72 987 (14.4)	0.05	2 639 (12.8)	10 460 (12.7)	0.00
H	2 118 (10.3)	56 311 (11.3)	0.03	2 110 (10.3)	8 303 (10.1)	0.01
**Index date**						
2 005–2006	3 207 (15.5)	54 929 (11.0)	0.13	3 179 (15.5)	12 825 (15.6)	0.00
2 007–2009^c^	2 926 (14.2)	55 322 (11.1)	0.09	2 912 (14.2)	11 755 (14.3)	0.00
2 009^c^–2011^c^	2 773 (13.4)	55 528 (11.1)	0.07	2 752 (13.4)	10 735 (13.1)	0.01
2 011^c^–2013^c^	2 668 (12.9)	55 718 (11.1)	0.06	2 650 (12.9)	10 772 (13.1)	0.01
2 013^c^–2015^c^	2 202 (10.7)	55 349 (11.1)	0.01	2 192 (10.7)	9 023 (11.0)	0.01
2 015^c^–2017^c^	1 955 (9.5)	56 096 (11.2)	0.06	1 946 (9.5)	7 602 (9.3)	0.01
2 017^c^–2019^c^	1 792 (8.7)	55 531 (11.1)	0.08	1 784 (8.7)	7 100 (8.6)	0.00
2 019^c^–2021^c^	1 666 (8.1)	55 596 (11.1)	0.10	1 659 (8.1)	6 630 (8.0)	0.00
2 021^c^–2023	1 473 (7.1)	55 931 (11.2)	0.14	1 470 (7.2)	5 761 (7.0)	0.01
**Comorbidities and risk factors**						
Ever smoker	7 099 (34.4)	152 932 (30.6)	0.08	7 046 (34.3)	27 625 (33.6)	0.01
Hypertension	3 767 (18.2)	75 621 (15.1)	0.08	3 738 (18.2)	14 303 (17.4)	0.02
Diabetes mellitus	2 502 (12.1)	45 471 (9.1)	0.10	2 484 (12.1)	9 557 (11.6)	0.01
Obesity	2 925 (14.2)	72 813 (14.6)	0.01	2 909 (14.2)	11 153 (13.6)	0.02
Hyperlipidaemia	4 975 (24.1)	114 742 (22.9)	0.03	4 937 (24.0)	19 277 (23.5)	0.01
Family history of CVDs	455 (2.2)	10 345 (2.1)	0.01	452 (2.2)	1 656 (2.0)	0.01
Alcohol abuse	348 (1.7)	3 414 (0.7)	0.09	344 (1.7)	1 160 (1.4)	0.02
Drug abuse	163 (0.8)	2 928 (0.6)	0.02	161 (0.8)	621 (0.8)	0.00
Cardiac valvular disease	668 (3.2)	15 915 (3.2)	0.00	665 (3.2)	2 469 (3.0)	0.01
Vascular disease	525 (2.5)	11 499 (2.3)	0.01	515 (2.5)	2 037 (2.5)	0.00
Atrial fibrillation	568 (2.7)	13 037 (2.6)	0.01	561 (2.7)	2 163 (2.6)	0.00
Ischaemic heart disease	1 415 (6.8)	30 900 (6.2)	0.02	1 405 (6.8)	5 418 (6.6)	0.01
CHF	492 (2.4)	9 907 (2.0)	0.03	489 (2.4)	1 877 (2.3)	0.01
Prior stroke	606 (2.9)	12 434 (2.5)	0.02	592 (2.9)	2 319 (2.8)	0.01
Chronic renal failure	1 034 (5.0)	18 211 (3.6)	0.07	1 022 (5.0)	3 889 (4.7)	0.01
COPD	764 (3.7)	11 442 (2.3)	0.08	755 (3.7)	2 689 (3.3)	0.02
Malignancy	1 387 (6.7)	27 713 (5.5)	0.05	1 372 (6.7)	5 112 (6.2)	0.02
**Medication use**						
Antiplatelets	3 818 (18.5)	74 419 (14.9)	0.10	3 785 (18.4)	14 508 (17.7)	0.02
Anticoagulants	324 (1.6)	8 185 (1.6)	0.00	321 (1.6)	1 230 (1.5)	0.01
Statins	4 542 (22.0)	92 650 (18.5)	0.09	4 505 (21.9)	17 608 (21.4)	0.01
**Healthcare utilization**						
Faecal occult blood test	2 653 (12.8)	45 648 (9.1)	0.12	2 631 (12.8)	9 880 (12.0)	0.02
Pneumococcal vaccine	1 907 (9.2)	35 434 (7.1)	0.08	1 895 (9.2)	7 112 (8.7)	0.02
Influenza vaccine	4 169 (20.2)	96 811 (19.4)	0.02	4 144 (20.2)	15 916 (19.4)	0.02
Prior hospitalization	3 117 (15.1)	59 690 (11.9)	0.09	3 081 (15.0)	11 613 (14.1)	0.03

Abbreviations: CHF, congestive heart failure, COPD, chronic obstructive pulmonary disease; CVDs, cardiovascular diseases; SMD, standardized mean difference.

a Socioeconomic status had missing values in 7.9% of subjects before matching and in 12.9% after matching.

b District code had missing values in 0.3% of subjects before matching and in 1.1% after matching.

c Midyear intervals; years are divided with no overlap.

During the 1 105 834 person-years of follow-up, 2 189 subjects experienced a stroke (Table 2). HBV patients had a slightly higher incidence rate of overall stroke compared to controls (2.13 vs. 1.94 per 1 000 person-years), yielding a non-statistically significant HR of 1.09 (95% CI 0.98–1.22). For ischaemic stroke, we observed 1 944 events with identical incidence rates in both groups (1.76 per 1 000 person-years) with a HR of 1.01 (95% CI 0.89–1.14). However, HBV was significantly associated with increased risk of ICH: with a total of 245 events, the incidence rate was nearly twice as high in the HBV group compared to non-HBV controls (0.37 vs. 0.19 per 1 000 person-years), resulting in a statistically significant HR of 1.82 (95% CI 1.35–2.45).

**Table 2.** Descriptive statistics, incidence rates, and hazard ratios (HRs) for the association between hepatitis B virus (HBV) and study outcomes (*N* = 102 720) Table 2. long description.

	No. of events	Follow-up duration (person-years)	Incidence rate (per 1 000 person-years)	HR (95% CI)
**Overall stroke^a^**				
HBV (*n* = 20 544)	472	221 608	2.13	1.09 (0.98–1.22)
Non-HBV (*n* = 82 176)	1 717	884 226	1.94	Reference
**Ischaemic stroke**				
HBV (*n* = 20 544)	391	221 608	1.76	1.01 (0.89–1.14)
Non-HBV (*n* = 82 176)	1 553	884 226	1.76	Reference
**Intracerebral haemorrhage (ICH)**				
HBV (*n* = 20 544)	81	221 608	0.37	1.82 (1.35–2.45)
Non-HBV (*n* = 82 176)	164	884 226	0.19	Reference

Abbreviation: CI, confidence interval.

a Overall stroke was defined as the composite outcome of ischaemic and intracerebral haemorrhagic (ICH) strokes.

The results were consistent across the sensitivity analyses: among subjects without a prior history of stroke (Supplementary Table S1), after excluding subjects diagnosed with HCV during follow-up (Supplementary Table S2), and when limiting the analysis to confirmed chronic HBV cases defined by at least two positive HBsAg tests obtained ≥6 months apart (Supplementary Table S3).

**Figure 2.**
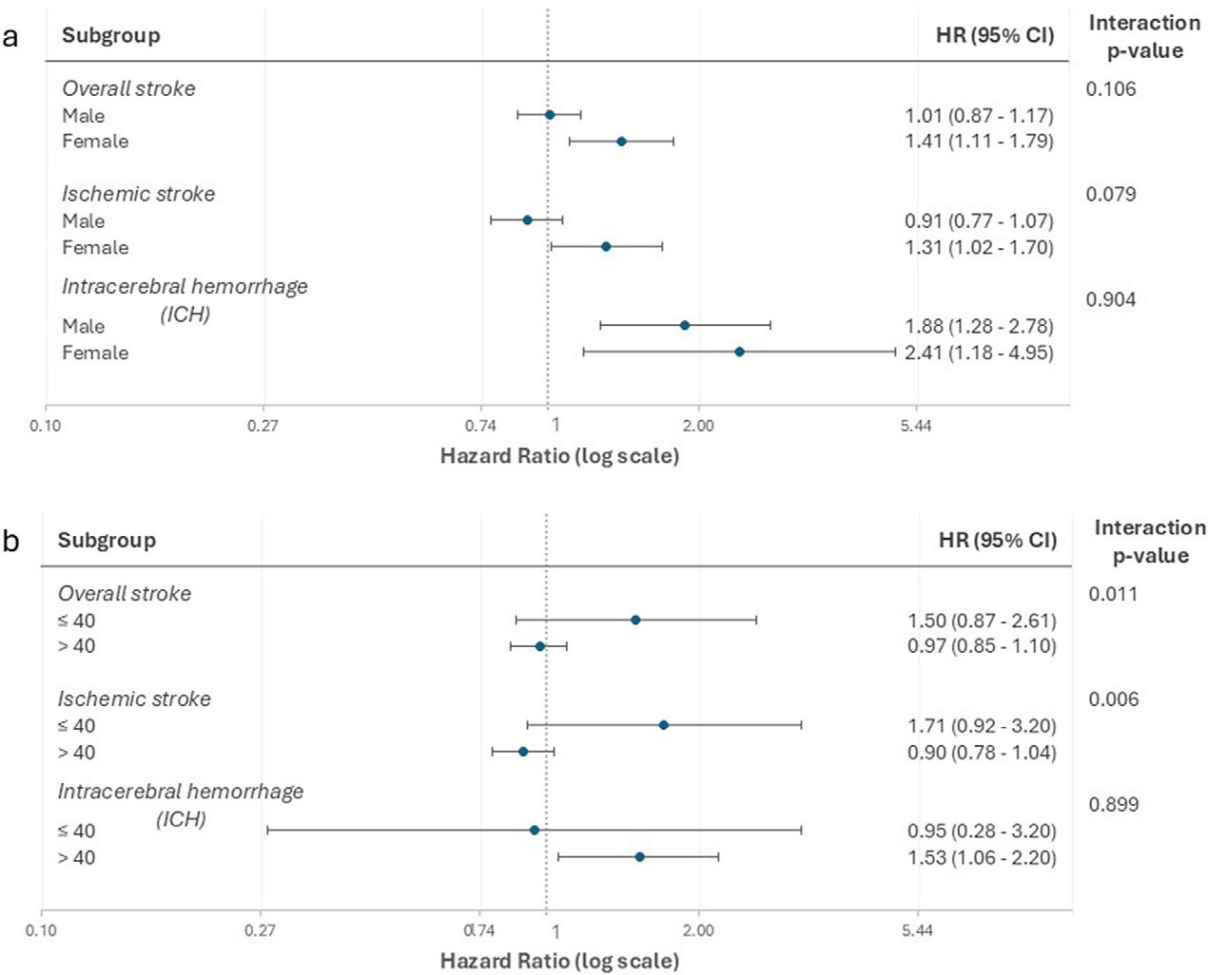
Forest plots for the association between hepatitis B virus (HBV) and overall stroke*, ischaemic stroke, and intracerebral haemorrhage (ICH), showing the hazard ratios (HRs) from the subgroup analyses by: (a) sex and by (b) median age. * Overall stroke was defined as the composite outcome of ischaemic and ICH strokes. Figure 2. long description.

Subgroup analysis stratified by median age and sex showed an increased risk of ischaemic stroke in subjects younger than 40 years (P for interaction = 0.006) and in females (P for interaction = 0.079) (Figure 2).

The results remained unchanged when HBV infection was accompanied by an elevated ALT level (Supplementary Table S4). An exploratory analysis showed that HBV with HDV coinfection was associated with increased risk of ischaemic stroke with an HR of 1.83 (95% CI, 1.00–3.34), whereas HBV without HDV infection was not associated with increased risk of ischaemic stroke with an HR of 0.99 (95% CI, 0.87–1.12) (Table 3).

**Table 3.** Descriptive statistics, incidence rates, and hazard ratios (HRs) for the association between HBV infection with and without hepatitis D virus (HDV) coinfection and study outcomes (*N* = 102 720) Table 3. long description.

	No. of events	Follow-up duration (person-years)	Incidence rate (per 1 000 person-years)	HR (95% CI)
**Overall stroke^a^**				
HBV/HDV (*n* = 657)	21	7 027	2.99	1.81 (1.03–3.19)
HBV/Non-HDV (*n* = 19 887)	451	214 581	2.1	1.07 (0.96–1.20)
Non-HBV^b^ (*n* = 82 176)	1 717	884 226	1.94	Reference
**Ischaemic stroke**				
HBV/HDV (*n* = 657)	19	7 027	2.7	1.83 (1.00–3.34)
HBV/Non-HDV (*n* = 19 887)	372	214 581	1.73	0.99 (0.87–1.12)
Non-HBV^b^ (*n* = 82 176)	1 553	884 226	1.76	Reference
**Intracerebral haemorrhage (ICH)**				
HBV/HDV (*n* = 657)	2	7 027	0.28	1.68 (0.30–9.31)
HBV/Non-HDV (*n* = 19 887) Non-HBV^b^ (*n* = 82 176)	79 164	214 581 884 226	0.37 0.19	1.82 (1.34–2.47) Reference

Abbreviation: CI, confidence interval.

a Overall stroke was defined as the composite outcome of ischaemic and intracerebral haemorrhagic (ICH) strokes.

b The non-HBV category is distinct from each HBV category, corresponding to the original propensity score-matched groups.

## Discussion

Our results show no significant association between HBV infection and overall stroke or ischaemic stroke. However, HBV infection was associated with a significantly increased risk of ICH. These findings remained robust across sensitivity analyses. Subgroup analysis further suggests that the association between HBV and ischaemic stroke risk is more pronounced and increased in subjects younger than 40 years and in females. In addition, HBV infection in the presence of HDV coinfection was associated with increased risk of ischaemic stroke.

Our results align with a large cohort study based on prospective biobank databases that found no association between HBV and overall stroke or ischaemic stroke, but identified an increased risk for ICH with an HR of 1.28 (95% CI, 1.06–1.55) [5]. Consistent with our findings, these results remained robust in a sensitivity analysis restricted to subjects without a history of prior cardiovascular disease. However, unlike our study, the study found no interaction effects for age or sex [5]. Similarly, a Korean study using a prospective biobank of male public servants reported an increased risk of ICH with an adjusted HR of 1.33 (95% CI, 1.15–1.53) [10]. In contrast to our findings, this study reported a decreased risk for ischaemic stroke with an adjusted HR of 0.79 (95% CI, 0.68–0.90) [10]. A decreased risk of ischaemic stroke was also reported in two retrospective cohort studies from Taiwan, which were based on insurance claims [11, 19]. Kuo et al. [19] found an adjusted HR of 0.61 (95% CI, 0.56–0.67), along with reduced mortality risk. Tseng et al. [11], using the same database but focusing specifically on diabetic patients, reported an adjusted HR of 0.77 (95% CI, 0.66–0.89) [11]. These contradictory results may stem from methodological limitations, including the use of diagnostic codes rather than laboratory confirmation for HBV exposure and the limited availability of potential confounders in claims data [11, 19]. Alternatively, these differences might reflect unique characteristics of the Taiwanese study population [20].

Biologically, chronic HBV infection can lead to an increased risk of ICH through three interrelated pathways. First, thrombocytopenia is common in chronic HBV infection, often occurring in early stages even before the onset of cirrhosis [7, 9]. This reduction in platelet count is associated with hepatic dysfunction, as demonstrated in both clinical and Mendelian randomization studies [7, 9]. HBV also directly affects bone marrow, further contributing to low platelet counts and increasing bleeding risk [5, 8, 10]. Second, impaired synthesis of coagulation factors occurs as liver function deteriorates, resulting in a complex, unstable haemostatic balance that predisposes to haemorrhagic events [1, 8, 21]. Third, emerging evidence suggests that chronic HBV infection may contribute to vascular fragility and endothelial dysfunction through sustained pro-inflammatory states and oxidative stress, potentially weakening cerebral vessels [9]. Chronic HBV infection is also linked with lower serum lipid levels, including cholesterol, likely due to altered hepatic function and changes in lipid metabolism, which may further increase the risk for ICH [5, 22]. However, the impact of these metabolic changes on atherogenesis or ischaemic stroke remains uncertain and may vary by sex and age [5, 7, 22, 23].

In our study, about 3.2% of HBV patients were coinfected with HDV, consistent with previous reports from Israel [24]. Our analysis showed that HBV subjects coinfected with HDV were at increased risk for ischaemic stroke. Chronic coinfection with HDV is characterized by more aggressive liver disease, faster progression to cirrhosis, and a stronger, more sustained inflammatory response [12]. Sustained inflammation is independently associated with increased ischaemic stroke risk [7, 12]. However, direct evidence linking HDV to stroke remains lacking.

Our study faces several limitations. First, as an observational study based on an administrative database, residual confounding remains a concern, since we could not control for information not coded in patients’ files, such as personality traits, lifestyle habits, and general health conditions that may be associated with both HBV activity and stroke development. However, to address concerns raised in previous studies regarding inadequate model adjustment, we employed propensity score-matching based on numerous potential confounders, including sociodemographic factors, cardiovascular comorbidities, cardiovascular risk factors, selected medications, chronic diseases, and healthcare utilization markers.

Second, regarding the potential for misclassification bias, we based the HBV exposure assessment on HBsAg laboratory test results. HBsAg testing is highly sensitive and specific and is considered the cornerstone of HBV diagnosis and screening [25]. In contrast, previous studies relied on point-of-care rapid diagnostic tests, which have lower validity [5] or ICD diagnostic codes [11, 19]. Moreover, the diagnosis of ICH was found to be valid within our database [13]. Nevertheless, misclassification bias remains a concern, and the expected non-differential misclassification would bias the results towards the null, although unlikely enough to explain the results for overall and ischaemic strokes. We also recognize that subjects with negative HBsAg could acquire HBV infection during follow-up. However, this risk is minimal given Israel’s low HBV incidence (~1–2/100 000 annually in adults) [17].

Third, we did not relate our findings to antiviral treatments or the severity of chronic HBV infection, although we performed an analysis by ALT baseline levels as a proxy marker for inflammation activity. However, we acknowledge that ALT is a nonspecific marker that may be elevated for reasons other than HBV [26].

Fourth, due to our reliance on ICD coding, we were unable to specifically address ischaemic stroke aetiologies. Future studies could explore whether HBV differentially affects particular stroke mechanisms.

Finally, although our results pertain to a specific healthcare system, the CHS covers more than 50% of the Israeli population, including diverse groups.

In conclusion, our findings suggest that HBV is not associated with an increased risk of overall or ischaemic stroke but is significantly associated with a higher risk of ICH. Yet, this study suggests a higher risk for ischaemic stroke in specific subgroups, including females, younger patients, and chronic coinfection with hepatitis D. Further studies are needed to investigate the relationship between those BV and stroke, including its subtypes.

## Supporting information

10.1017/S095026882610154X.sm001 Yahav et al. supplementary material Yahav et al. supplementary material

## Data Availability

Individual-level data cannot be publicly available due to legal restrictions. All data relevant to this analysis were presented in the paper.

## Supplementary material

The supplementary material for this article can be found at http://doi.org/10.1017/S095026882610154X.

## [Figure 1] Long description

At the top, Clalit Health Services database is the source. Below, adults 20 years and older tested for HBsAg between 2005 and 2023 total 1,788,688. To the right, 6,580 with HBsAg positive before 2005 are excluded. The remaining are split into non-HBV (H BsAg negative, n equals 1,761,448) and HBV (H BsAg positive, n equals 20,660). The non-HBV group is randomly reduced to 500,000, then propensity score-matched to 82,176. The HBV group undergoes propensity score-matching 1 to 4, resulting in 20,544 matched individuals.

Navigate back to [Figure 1].

## [Table 1] Long description

The table contains seven columns. The first column lists variables, grouped as demographics, population sector, socioeconomic status, district, index date, comorbidities and risk factors, medication use, and healthcare utilization. For each variable, the next three columns show values for HBV group, Non-HBV group, and SMD before matching. The final three columns show HBV, Non-HBV, and SMD after matching. For example, age at index date is 44.6 plus or minus 15.8 years for HBV and 39.4 plus or minus 17.5 for Non-HBV before matching, SMD 0.31; after matching, 44.6 plus or minus 15.8 for HBV and 44.2 plus or minus 17.9 for Non-HBV, SMD 0.02. Sex (male) is 11,620 (56.2 percent) for HBV and 185,566 (37.1 percent) for Non-HBV before matching, SMD 0.39; after matching, 11,539 (56.2 percent) for HBV and 46,625 (56.7 percent) for Non-HBV, SMD 0.01. Population sector is divided into Jewish and Arab, with respective counts and percentages. Socioeconomic status is categorized as low, middle, and high. Districts are labeled A through H. Index date is divided into intervals from 2005–2006 through 2021–2023. Comorbidities include ever smoker, hypertension, diabetes mellitus, obesity, hyperlipidaemia, family history of CVDs, alcohol abuse, drug abuse, cardiac valvular disease, vascular disease, atrial fibrillation, ischaemic heart disease, CH F, prior stroke, chronic renal failure, COP D, and malignancy. Medication use includes antiplatelets, anticoagulants, and statins. Healthcare utilization includes faecal occult blood test, pneumococcal vaccine, influenza vaccine, and prior hospitalization. SMD values are generally reduced after matching, indicating improved balance between groups. Footnotes clarify missing data for socioeconomic status and district code, and define midyear intervals.

Navigate back to [Table 1].

## [Table 2] Long description

From top to bottom, the table has three main sections: overall stroke, ischaemic stroke, and intracerebral haemorrhage (ICH). Each section compares HBV and non-HBV groups. For overall stroke, HBV group (n equals 20 544) had 472 events, 221 608 person-years, incidence rate 2.13 per 1 000 person-years, hazard ratio 1.09 with 95 percent confidence interval 0.98 to 1.22. Non-HBV group (n equals 82 176) had 1 717 events, 884 226 person-years, incidence rate 1.94, hazard ratio reference. For ischaemic stroke, HBV group had 391 events, incidence rate 1.76, hazard ratio 1.01 with 95 percent confidence interval 0.89 to 1.14. Non-HBV group had 1 553 events, incidence rate 1.76, hazard ratio reference. For ICH, HBV group had 81 events, incidence rate 0.37, hazard ratio 1.82 with 95 percent confidence interval 1.35 to 2.45. Non-HBV group had 164 events, incidence rate 0.19, hazard ratio reference. Footnote states overall stroke is the composite of ischaemic and ICH strokes. Abbreviation C I stands for confidence interval.

Navigate back to [Table 2].

## [Figure 2] Long description

Panel a shows hazard ratios for overall stroke, ischemic stroke, and intracerebral hemorrhage by sex. The y-axis lists subgroups: overall stroke (male, female), ischemic stroke (male, female), and intracerebral hemorrhage (male, female). The x-axis is labeled hazard ratio (log scale) ranging from 0.10 to 5.44. For overall stroke, males have HR 1.01 (95 percent CI 0.87 to 1.17), females 1.41 (1.11 to 1.79), interaction p-value 0.106. For ischemic stroke, males HR 0.91 (0.77 to 1.07), females 1.31 (1.02 to 1.70), interaction p-value 0.079. For intracerebral hemorrhage, males HR 1.88 (1.28 to 2.78), females 2.41 (1.18 to 4.95), interaction p-value 0.904. Panel b shows the same outcomes by age group (less than or equal to 40, greater than 40). For overall stroke, less than or equal to 40 HR 1.50 (0.87 to 2.61), greater than 40 HR 0.97 (0.85 to 1.10), interaction p-value 0.011. For ischemic stroke, less than or equal to 40 HR 1.71 (0.92 to 3.20), greater than 40 HR 0.90 (0.78 to 1.04), interaction p-value 0.006. For intracerebral hemorrhage, less than or equal to 40 HR 0.95 (0.28 to 3.20), greater than 40 HR 1.53 (1.06 to 2.20), interaction p-value 0.899. Each data point is shown as a dot with horizontal lines for confidence intervals. The vertical dashed line at HR 1 indicates no effect.

Navigate back to [Figure 2].

## [Table 3] Long description

The table contains three main sections: overall stroke, ischaemic stroke, and intracerebral haemorrhage. For overall stroke, HBV/HDV group (n equals 657) had 21 events, 7027 person-years, incidence rate 2.99 per 1000 person-years, hazard ratio 1.81 with 95 percent confidence interval 1.03 to 3.19. HBV/Non-HDV (n equals 19887) had 451 events, 214581 person-years, incidence rate 2.1, hazard ratio 1.07 with 0.96 to 1.20. Non-HBV (n equals 82176) had 1717 events, 884226 person-years, incidence rate 1.94, reference hazard ratio. For ischaemic stroke, HBV/HDV had 19 events, incidence rate 2.7, hazard ratio 1.83 with 1.00 to 3.34. HBV/Non-HDV had 372 events, incidence rate 1.73, hazard ratio 0.99 with 0.87 to 1.12. Non-HBV had 1553 events, incidence rate 1.76, reference hazard ratio. For intracerebral haemorrhage, HBV/HDV had 2 events, incidence rate 0.28, hazard ratio 1.68 with 0.30 to 9.31. HBV/Non-HDV had 79 events, incidence rate 0.37, hazard ratio 1.82 with 1.34 to 2.47. Non-HBV had 164 events, incidence rate 0.19, reference hazard ratio. The non-HBV group is distinct from HBV groups. Overall stroke is defined as the composite of ischaemic and intracerebral haemorrhagic strokes.

Navigate back to [Table 3].
