# Supplementary material for: Association between chronic hepatitis B virus infection and stroke risk: a propensity score-matched analysis
Source: Epidemiol Infect. 2026 May 5;154:e66. doi: 10.1017/S095026882610154X (PMC13244349; doi:10.1017/S095026882610154X)
Supplement: Yahav et al. supplementary material [file S095026882610154Xsup001.docx]

**Supplementary Materials**

**Association between chronic hepatitis B virus infection and stroke risk: A propensity score-matched analysis**

Amir Yahav, MD, PhD^1,2*^, Doaa Ryan, MD^2*^, Nael Tuma, MD, MPH^3^, Anat Arbel, MD^4^, Nili Stein MPH^2,5^, Eitan Auriel, MD, MSc^6,7^, Walid Saliba, MD, MPH^1,2^

*Equal contribution

^1^Ruth and Bruce Rappaport Faculty of Medicine, Technion-Israel Institute of Technology, Haifa, Israel, ^2^Department of Community Medicine and Epidemiology, Lady Davis Carmel Medical Center, Haifa, Israel , ^3^Northern District Health Bureau, Ministry of Health, Nof Hagalil, Israel, ^4^Infectious Diseases Unit, Lady Davis Carmel Medical Center, Haifa, Israel, ^5^Statistical Unit, Lady Davis Carmel Medical Center, Haifa, Israel, ^6^Department of Neurology, Rabin Medical Center, Petach Tikva, Israel, and ^7^Gray Faculty of Medical & Health Sciences, Tel Aviv University, Tel Aviv, Israel.

**Short title:** Hepatitis B virus and stroke

**Supplementary Materials**

**Contents**

**Supplementary Table S1:** Descriptive statistics, incidence rates and hazard ratios (HRs) for the association between hepatitis B virus (HBV) and study outcomes: Analysis restricted to subjects without a prior history of stroke *(N=97,540)* ……………………………………………………………………….………..….**3**

**Supplementary Table S2:** Descriptive statistics, incidence rates and hazard ratios (HRs) for the association between hepatitis B virus (HBV) and study outcomes: Analysis excluding subjects diagnosed with hepatitis C virus (HCV) during follow-up *(N=101,673)*………………………….………..………….**4**

**Supplementary Table S3:** Descriptive statistics, incidence rates and hazard ratios (HRs) for the association between hepatitis B virus (HBV) and study outcomes: Analysis restricted to confirmed chronic HBV cases defined by $\geq$2 positive HBsAg tests obtained $\geq$6 months apart (N=52,150)………….**5**

**Supplementary Table S4:** Subgroup analysis by baseline alanine aminotransferase (ALT) levels of subjects with hepatitis B virus (HBV) ^‡^, for the association between HBV and study outcomes *(N= 98,730) ……………………………………………*…………………………………………………………………………………………………**6**

**Supplementary Table S1:** Descriptive statistics, incidence rates and hazard ratios (HRs) for the association between hepatitis B virus (HBV) and study outcomes: Analysis restricted to subjects without a prior history of stroke *(N=97,540)*

|  | **No. of events** | **Follow-up duration (person-years)** | **Incidence rate (per 1,000 person-years)** | ***HR (95% CI)*** |
| --- | --- | --- | --- | --- |
| **Overall stroke***  HBV (n=19,952)  Non-HBV (n=77,588) | 404  1,404 | 218,063  847,807 | 1.85  1.67 | 1.10 (0.97-1.25)  Reference |
| **Ischemic stroke**  HBV (n=19,952)  Non-HBV (n=77,588) | 331  1,269 | 218,063  847,807 | 1.52  1.50 | 1.01 (0.88-1.16)  Reference |
| **Intracerebral hemorrhage (ICH)**  HBV (n=19,952)  Non-HBV (n=77,588) | 73  135 | 218,063  847,807 | 0.35  0.16 | 1.87 (1.35-2.58)  Reference |

Abbreviations: CI, confidence interval

* Overall stroke was defined as the composite outcome of ischemic and intracerebral hemorrhagic (ICH) strokes.

**Supplementary Table S2:** Descriptive statistics, incidence rates and hazard ratios (HRs) for the association between hepatitis B virus (HBV) and study outcomes: Analysis excluding subjects diagnosed with hepatitis C virus (HCV) during follow-up *(N=101,673)*

|  | **No. of events** | **Follow-up duration (person-years)** | **Incidence rate (per 1,000 person-years)** | ***HR (95% CI)*** |
| --- | --- | --- | --- | --- |
| **Overall stroke***  HBV (n=20,403)  Non-HBV (n=81,270) | 469  1,694 | 219,647  873,065 | 2.14  1.94 | 1.10 (0.98-1.23)  Reference |
| **Ischemic stroke**  HBV (n=20,403)  Non-HBV (n=81,270) | 390  1,534 | 219,647  873,065 | 1.78  1.76 | 1.02 (0.9-1.15)  Reference |
| **Intracerebral hemorrhage (ICH)**  HBV (n=20,403)  Non-HBV (n=81,270) | 79  160 | 219,647  873,065 | 0.36  0.18 | 1.85 (1.37-2.50)  Reference |

Abbreviations: CI, confidence interval

* Overall stroke was defined as the composite outcome of ischemic and intracerebral hemorrhagic (ICH) strokes.

**Supplementary Table S3:** Descriptive statistics, incidence rates and hazard ratios (HRs) for the association between hepatitis B virus (HBV) and study outcomes: Analysis restricted to confirmed chronic HBV cases defined by $\geq$2 positive HBsAg tests obtained $\geq$6 months apart (N=52,150)

|  | **No. of events** | **Follow-up duration (person-years)** | **Incidence rate (per 1,000 person-years)** | ***HR (95% CI)*** |
| --- | --- | --- | --- | --- |
| **Overall stroke***  HBV (n=10,430)  Non-HBV (n=41,720) | 248  855 | 131,440  474,654 | 1.89  1.80 | 0.97 (0.83-1.14)  Reference |
| **Ischemic stroke**  HBV (n=10,430)  Non-HBV (n=41,720) | 205  774 | 131,440  474,654 | 1.56  1.63 | 0.89 (0.75-1.05)  Reference |
| **Intracerebral hemorrhage (ICH)**  HBV (n=10,430)  Non-HBV (n=41,720) | 43  81 | 131,440  474,654 | 0.33  0.17 | 1.76 (1.17-2.65)  Reference |

Abbreviations: CI, confidence interval

* Overall stroke was defined as the composite outcome of ischemic and intracerebral hemorrhagic (ICH) strokes.

**Supplementary Table S4:** Subgroup analysis by baseline alanine aminotransferase (ALT) levels of subjects with hepatitis B virus (HBV) ^‡^, for the association between HBV and study outcomes *(N= 98,730)*

|  | **No. of events** | **Follow-up duration (person-years)** | **Incidence rate (per 1,000 person-years)** | ***HR (95% CI)*** |
| --- | --- | --- | --- | --- |
| **Overall stroke***  HBV / *ALT > 40* U/L (n=4,734)  HBV / *ALT ≤ 40* U/L (n=15,012)  Non-HBV^§^ (n=78,984) | 101  368  1,676 | 50,719  160,895  846,734 | 1.99  2.29  1.98 | 1.00 (0.80-1.27)  1.16 (1.02-1.32)  Reference |
| **Ischemic stroke**  HBV / *ALT > 40* U/L (n=4,734)  HBV / *ALT ≤ 40* U/L (n=15,012)  Non-HBV^§^ (n=78,984) | 83  305  1,519 | 50,719  160,895  846,734 | 1.64  1.90  1.79 | 0.92 (0.71-1.19)  1.01 (0.93-1.23)  Reference |
| **Intracerebral hemorrhage (ICH)**  HBV / *ALT > 40* U/L (n=4,734)  HBV / *ALT ≤ 40* U/L (n=15,012)  Non-HBV^§^ (n=78,984) | 18  63  157 | 50,719  160,895  846,734 | 0.35  0.39  0.19 | 1.78 (0.95-3.35)  1.96 (1.39-2.77)  Reference |

Abbreviations: HR, hazard ratio; CI, confidence interval

^‡^ A total of 3,990 HBV patients had missing baseline ALT test results.

* Overall stroke was defined as the composite outcome of ischemic and intracerebral hemorrhagic (ICH) strokes.

§ The non-HBV category is distinct for each HBV category, corresponding to the original propensity score-matched groups.
